# Distinctive Clinico-Pathological Characteristics of Colorectal Cancer in Sabahan Indigenous Populations

**DOI:** 10.31557/APJCP.2021.22.3.749

**Published:** 2021-03

**Authors:** Anuradha Valan, Fatimah Najid, Pradeep Chandran, Azuwani Binti Abd Rahim, Jitt Aun Chuah, April Camilla Roslani

**Affiliations:** 1 *Department of General Surgery, Queen Elizabeth Hospital, Kota Kinabalu, Sabah, Malaysia.*; 2 *Department of Surgery, Faculty of Medicine, University of Malaya, Malaysia. *; 3 *Department of Surgery, Duchess of Kent Hospital, Sandakan, Sabah, Malaysia. *; 4 *Department of Surgery, Tawau Hospital, Sabah, Malaysia. *

**Keywords:** Colorectal cancer, colorectal carcinoma, indigenous, Sabah

## Abstract

**Background:**

Malaysia is an ethnically diverse nation, comprising Malay, Chinese, Indian and indigenous groups. However, epidemiological studies on colorectal cancer have mainly focused on the three main ethnic groups. There is evidence that the clinico-pathological characteristics of some cancers may differ in indigenous populations, namely that they occur earlier and behave more aggressively. We aimed to determine if there were similar differences in colorectal cancer, focusing on the indigenous populations of Sabah.

**Methods:**

Histopathological reports of all patients diagnosed with colorectal carcinoma from January 2012 to December 2016 from public hospitals in Sabah were retrieved from the central computerized database of the Pathology Department of Queen Elizabeth Hospital in Kota Kinabalu, Sabah. Supplementary data was obtained from patients’ case files from each hospital. Clinico-pathological data were analysed using the IBM SPSS Statistical Software Version 23 for Windows for descriptive statistics (mean, median, ASR, AR, relative risk) and inferential statistics (Chi square test).

**Results:**

A total of 696 patients met the inclusion criteria. The median age for colorectal cancer in Sabah was 62 years (95% CI 60.3 to 62.3), with an age specific incidence rate of 21.4 per 100 000 population. The age specific incidence rate in the indigenous populations was 26.6 per 100 000, much lower than the Chinese, at 65.0 per 100 000. The risk of colorectal cancer occurring before the age of 50 was three times higher in the indigenous population compared to the Chinese. The tumours were mainly left-sided (56.5%), adenocarcinoma in histology (98.4%) and moderately differentiated (88.7%). Approximately 79.2% of patients received curative treatment.

**Conclusion:**

Indigenous populations in Sabah develop colorectal cancer at an earlier age, and present at more advanced stages. This has implications for screening and therapeutic strategic planning.

## Introduction

Colorectal cancer (CRC) is emerging as one of the leading causes of morbidity and mortality worldwide. While disease burden is largely concentrated in countries with high and very high Human Development Index (HDI), the incidence in low HDI countries is rising, with significant socioeconomic consequences (Bray et al., 2012; Bray et al.,2018; Arnold et al., 2017) .

Malaysia is a medium HDI country with a diverse population. It is divided into Peninsular and East Malaysia, separated by the South China Sea (Jabatan Perangkaan Malaysia, 2018). The population is estimated to be 32.4 million, with an annual growth rate of 1.1 percent, and a median age of 28.3 years (Jabatan Perangkaan Malaysia, 2018). The dominant ethnic group is the Bumiputra (Malays and indigenous populations: 69.1%), followed by Chinese (23.0%), Indians (6.9%) and others (1.0%) (Jabatan Perangkaan Malaysia, 2018).

Malaysia’s Age-Standardized Rate (ASR) for CRC is much lower than that of high-HDI countries like the United States of America (18.3 versus 45.9 per 100 000)( Hassan et al., 2014; Azizah et al., 2016). However, there is great variation between different ethnic groups. It is highest in the Chinese (21.1 versus 17.1 per 100 000 in males versus females) (Hassan et al., 2014; Azizah et al., 2016), and in Penang, which has a predominantly Chinese population (23.3 versus 18.7 per 100 000 in males versus females (Hassan et al., 2014; Azizh et al., 2016)).

However, national cancer reports are skewed by Peninsular data. Although lower mean age than the national average has been reported for CRC in East Malaysia and indigenous populations, the numbers captured were small, and might not be representative (Qureshi et al., 2001). 

Sabah is the second largest state in Malaysia by land mass, but has only 3.9 million inhabitants, comprising predominantly indigenous populations (83.7%) (Jabatan Perangkaan Malaysia, 2018). Reported CRC incidence was lower than the national average, but ethnicities in national reports were only classified as Malays, Chinese, Indians and others (including foreigners) ( Hassan et al., 2014; Azizah et al., 2016). It is clear that data truly representative of indigenous populations is lacking.

This data is essential in order to rationalize screening, create public health awareness, improve access to healthcare and optimize outcomes. Uniquely, pathology services in Sabah are centralized to a single public hospital, thus providing potentially comprehensive capture of all diagnosed CRCs. 

## Materials and Methods

Malaysians in Sabah diagnosed with CRC by histopathology, including synchronous and metachronous tumours, from January 2012 to December 2016, were retrospectively analyzed. Recurrent CRCs, non-Malaysians and colorectal lymphomas were excluded. 

Clinico-pathological data was obtained from the Department of Pathology, Queen Elizabeth Hospital, Kota Kinabalu, the state hospital which analyzes the histopathological specimens from all public hospitals in Sabah. Supplementary data was obtained from case notes provided by the referring hospitals. 

This study was done in accordance with the Declaration of Helsinki and the ethical principles of the Good Clinical Practice framework. It was approved by the Medical Research Ethics Committee (MREC), with the following ethics approval number MREC ID NO NMRR-16-1496-31925.

*Statistical analysis, statistical methods and statistical terms*

The data was analysed using descriptive and inferential statistics (IBM SPSS Statistical Software Version 23 for Windows). Baseline demographic data was expressed as frequency, median, mean and percentages for continuous data or frequency and percentages for categorical data. Incidence rates and mortality rates were calculated using the age standardised, age specific rates and age specific mortality rates, with either World Health Organisation Standard Population 2001(WHO, 2001) or Sabah Population Estimate 2012 (Department of Statistics Malaysia, 2018) as the denominators. Cross tabs and Chi Square test were used to analyse the relationship of ethnicity and age with the incidence colorectal carcinoma. A p-value of less than 0.05 was considered statistically significant. The definition of the statistical terms used are summarised in the appendices. 

## Results

**Figure 1:**
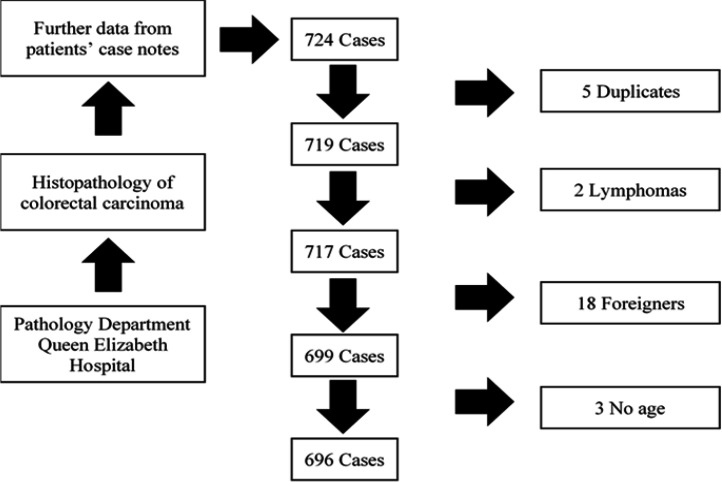
Colorectal Cancer in Sabah 2012 to 2016: Consort Diagram

Contributing hospitals were Queen Elizabeth Hospital (478), Tawau Hospital (92), Duchess of Kent Hospital (79), Beaufort Hospital (1), Keningau Hospital (25), Kudat Hospital (2), Lahad Datu Hospital (6) and Labuan Hospital (13). Of the 724 cases, five were duplicates, two were colon lymphomas, 18 were foreigners and three had no documented age, leaving a total of 696 cases for analysis (Figure 1) (Table 1). CRC incidence exceeded 100 every year, but was highest in 2012 and lowest in 2016. The latter may be due to delayed data entry of cases diagnosed in late 2016. 

Overall, age and gender distribution were similar to the national average. However, most CRCs occurred in the indigenous population (486, 69.8%), followed by the Chinese (195, 28.0%), Malays (13, 1.9%) and Indians (2, 0.3%, Table 1). Hence, we focused our sub-analyses on the indigenous and Chinese populations. 

The median age for CRC in the indigenous population was seven years younger than the Chinese (Table 2). While the majority of patients were over the age of 50 years (`Old’: 571, 82.0%), a larger proportion of the indigenous population were below the age of 50 years (`Young’: 107, 22.0%) (Table 2). 

*Age specific incidence rate*

Overall, the age specific incidence rate was highest in Chinese males, at 77.3 per 100,000 population (Table 1). However, in the `young’, the indigenous population had the highest rate, at 6.7 per 100,000 population (Table 2). In general, both groups showed and increasing incidence after the age of 50 years (Table 2).

*Age standardized incidence rate*

**Figure 2:**
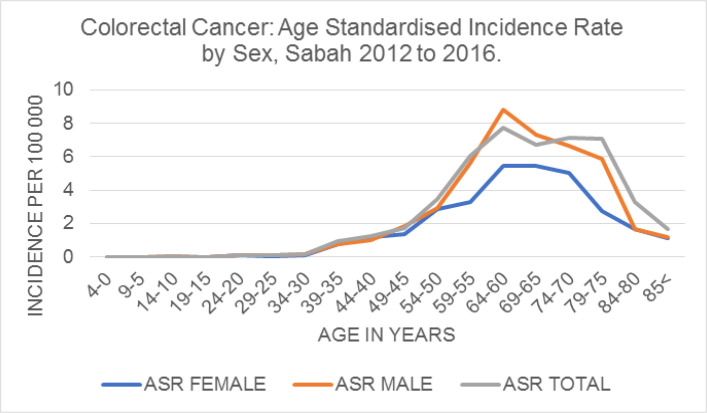
Colorectal Cancer in Sabah 2012 to 2016: Age standardised incidence rate per 100,000 population World Health Organisation Standard Population

The age standardized incidence rate was 21.4 per 100, 000 with a peak at 60 to 79 years, and a steady decline thereafter (Table 1, Figure 2). It was higher in males (23.3 per 100,000) than females (16.9 per 100,000) with a peak incidence at 60 to 64 years (Table 1, Figure 2). There was an increasing incidence from 45 years, then a steady decline after the age of 74, in both sexes (Figure 2). 

*Age specific mortality rate*

**Figure 3:**
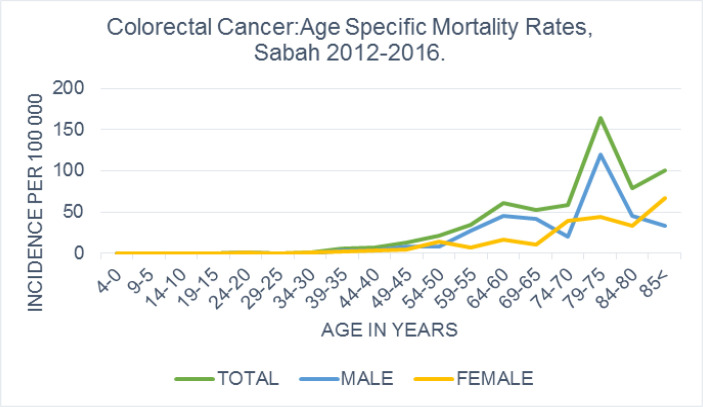
Colorectal Cancer in Sabah 2012 to 2016: Age Specific Mortality Rate Per 100 000 Sabah Population

Two hundred and three patients died during the period of study (29.2%, Table 1), for an age specific mortality rate of 6.2 per 100,000. Sixty-two percent of the mortalities were males, who had a higher mortality rate than females (3.9 versus 2.4 per 100,000, Table 1). The overall age specific mortality rates peaked at 75 to 79 years for both males and females, steadily decreasing thereafter (Figure 3). 

Mortality was higher in the Chinese than the indigenous population, (20.0 versus 7.5 per 100,000). While mortality in `young’ patients was similar in both ethnic groups, there was a distinct difference in the `old’ patients, with the Chinese having nearly twice the mortalities (81.5 vs 43.3 per 100,000) (Table 2). 

*Age standardized mortality rate*

**Figure 4:**
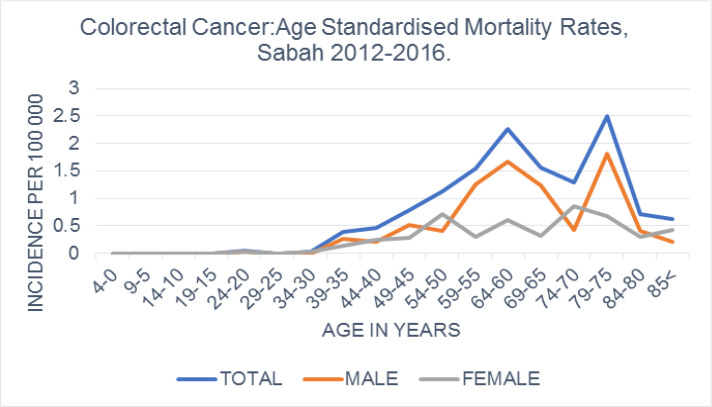
Age Standardised Mortality Rate Per 100,000 World Health Organisation Standard Population

The age standardised mortality rate was 6.2 per 100,000, and was higher in males compared to females (3.9 per 100 000 vs 2.4 per 100 000 population; Table 1). It showed two peaks at the age of 60 to 64 and 75 to 79 respectively (Figure 4).

*Relative Risk*

`Young’ and `old’ cohorts in the indigenous and Chinese ethnic groups were sub-analysed. The indigenous group had a relative risk of 3.1 for developing CRC at an earlier age compared to the Chinese, and this was statistically significant (Table 2).

*Clinical Characteristics and Treatment of Colorectal Cancer in Sabah*

*Location, Histology, Grading, Staging*

CRCs were predominantly left-sided (56.5 %), similar to national and global reports (Goh et al., 2005; Hassan et al., 2014; Lee et al., 2015). Nearly all were adenocarcinomas (98.4%) of moderately differentiated grade (88.7%), with no significant differences between the indigenous and non- indigenous populations. The majority were stage II and III (12.9% and 15.1% respectively), but more than half did not have documented staging. 

*Treatment*

The majority of CRCs who underwent treatment had curative surgery (79.1%), and this was similar in all ethnic groups. The rest had some form of palliative management or refused treatment entirely. Approximately 19.1% of patients received chemotherapy, while 4.5% were offered, but refused. Only 5.6% underwent radiotherapy. We were unable to make any inferences regarding ethnic differences in terms of cancer treatments due to the large amount of missing data. 

**Table 1:** Colorectal Cancer in Sabah 2012 to 2016: Mean, Median, Incidence and Mortality Rates by Sex

Age/sex	Male (n)	Female (n)	Total (n)
Frequency	418	278	696
Percentage	60.1	39.9	100
Median	62	62	62
Interquartile range	18	20	18
Confidence interval 95%	53.2-55.6	59.5-62.7	60.3-62.3
Ethnicity/sex	Male n (%)	Female n (%)	Total n (%)
Indigenous	291 (41.8)	195 (28.0)	486 (69.8)
Chinese	120 (17.2)	75 (10.8)	195 (28.0)
Malay	5 (0.7)	8 (1.1)	13 (1.9)
Indian	2 (0.3)	0 (0)	2 (0.3)
Incidence rates	n (%) (ar)	n (%) (ar)	n (%) (ar)
Indigenous	291(41.8) (31.5)	195 (28.0) (21.5)	486 (69.8) (26.6)
Chinese	120 (17.2) (77.3)	75 (10.8) (51.9)	195 (28.0) (65.0)
Malay	5 (0.7) (4.1)	8 (1.1) (6.8)	13 (1.9) (5.4)
Indian	2 (0.3) (34.5)	0 (0) (0)	2 (0.3) (18.2)
Total ar	418 (60.1) (23.3)	278 (39.9) (16.9)	696 (100) (21.4)
Total asr	418 (60.1) (23.3)	278 (39.9) (16.9)	696 (100) (21.4)
Mortality rates	n (%) (ar)	n (%) (ar)	n (%) ar
Ar (mortality)	126 (62.1) (3.9)	77 (37.9) (2.4)	203 (29.2) (6.2)
Asr (mortality)	126 (62.1) (3.9)	77 (37.9) (2.4)	203 (29.2) (6.2)

**Table 2:** Colorectal Cancer in Sabah 2012 to 2016: Mean, median, mortality rates, incidence rates, mortality rates and relative risks by ethnicity

Age/ethnicity	Indigenous (n)	Chinese (n)	Malay (n)	Indian (n)
Frequency	486	195	13	2
Median	60	67	62	47
Interquartile range	19	17	20	0
Mean	59.3	66.3	61.1	47
Standard deviation	13.4	12	12.7	14.1
Confidence interval 95%	58.1-60.5	64.6-68	53.4-68.8	-254.3
Incidence rates	n (%) (ar)	n (%) (ar)	n (%) (ar)	n (%) (ar)
Ar	486 (69.8) (26.6)	195 (28.0)(65.0)	13 (1.9)(5.4)	2(0.3) (18.2)
Mortality rates	n (%) (ar)	n (%) (ar)	n (%) (ar)	n (%) (ar)
Ar (mortality)	137 (67.5) (7.5)	60 (29.6) (20)	6 (3.0) (2.5)	203 (29.2) (6.2)
Incidence rates	n (ar)	n (ar)	n (ar)	n (ar)
Age < 50 years	107 (6.7)	14 (6)	3 (1.4)	1 (10.2)
Age > 50 years	379 (170.8)	181 (273)	10 (49.5)	1 (83.3)
Mortality rates	n (ar)	n (ar)	n (ar)	n (ar)
Age < 50 years	41 (2.6)	6 (2.57)	1 (0.5)	0 (0)
Age > 50 years	96 (43.3)	54 (81.5)	5 (24.8)	0 (0)
Relative risks	n (%)	n (%)	Relative risks	
Age < 50 years	107 (22)	14 (7.2)	3.1 (ci 1.8 - 5.2) p< 0.0001	
Age > 50 years	379 (78)	181 (92.8)	0.8 (0.8 - 0.9) p < 0.0001	

## Discussion

Worldwide, differences in cancer incidence and survival between indigenous and non-indigenous populations have been reported (Dachs et al., 2008; Moore et al., 2015) Suggested causes for poorer cancer survival in indigenous populations are late diagnosis and poor access to treatment ( Hill et al., 2013; Moore et al., 2014).

However, there may also be intrinsic factors. The Maori have been reported as developing CRC at a younger age, with more co-morbidities and acute presentations (Hill et al., 2010; Hill et al., 2010; Swart et al., 2013). The indigenous population in North Queensland, Australia is more likely to have poorly differentiated adenocarcinomas (Lu et al., 2005).

In South East Asia, Brunei Darussalam, which has geo-ethnic similarities to Malaysia, and has close proximity to Sabah, reported that the proportion of young CRCs was highest amongst their indigenous population (30.8%, versus 10% in the Chinese) (Chong et al., 2009; Quammen., 2013; Koh et al., 2015) .

Sabah has the highest concentration of indigenous populations in Malaysia, and our study is the first to report the detailed clinico-pathological characteristics of the largest Malaysian indigenous CRC cohort analysed thus far (Hassan et al., 2014; Abu Hassan et al., 2016; Azizah et al., 2016).

Interestingly, the age standardized rate of CRC in Sabah is comparable to high-HDI regions such as Europe and Australasia, and higher than the national rate ( Hassan et al., 2014; Azizah et al., 2016; Bray et al., 2018). 

Despite this, the overall age specific mortality rate for Sabah was lower than the national rate. It was also low in the indigenous population. It may be that some deaths were not reported as cancer deaths - in Sabah, the commonest reported cause of death is road traffic accidents (Theebalakshmi Kunasekaran, 2017). It is also possible that patients sought treatment elsewhere, presumably colorectal centres in Peninsular Malaysia or nearby Asian countries, and subsequently died there.

On the other hand, the age specific mortality rate for the Sabah Chinese population was markedly high, as compared to the national Chinese population (Hassan et al., 2014). Contributing factors would include an older population, more co-morbidities, and non-cancer deaths. Proportionally, the Chinese form only 8.7% of the Sabah population, of which 22.1% are above 50 years; thus any mortalities would be greatly impact the rate.

Unfortunately, there is still poor acceptance of treatment in a significant proportion of patients. Access to healthcare may be one obstacle. Sabah is a state that is still largely rural (Yan, 2007; Mansur and Kogid, 2008). The transportation system in rural areas is under-developed, limiting physical access to tertiary hospitals ( Yan, 2007; Mansur and Kogid, 2008; Lim et al., 2011). Furthermore, Sabah has a high poverty rate, thus patients may not be able to afford the cost of treatment (Mansur and Kogid, 2008). 

Cultural norms may also influence the acceptance of treatments. The indigenous people are a very tight-knit community, especially in rural areas, living in long houses as “one big happy family”. Duty to the community is a top priority, as enshrined in the social concepts of “gemeinschaft and gesellschaft”. Beliefs that treatments are harmful can be propagated within the community, making certain treatment is refused without a particular reason. The “farming culture” prioritizes work over health. Stoicism leads them to only seek treatment for disease at its worst stage or even refuse treatment all together (Strasser, 2003).

Nevertheless, there are acknowledged gaps in healthcare delivery, in terms of expertise and resources. At present, CRCs in Sabah are predominantly managed by general surgeons. Medical oncology services in the public sector are limited to a single centre in Likas, and radiation oncology is not widely available (Lim et al., 2011). Thus, there are challenges to establishing a focused CRC service, without appropriate support from the government. Although surgical treatment is expensive, addressing the cancer earlier with better expertise and tools leads to lower costs in the long run, as the need for more expensive and prolonged therapies when the cancer in more advanced can be avoided (NICE guideline, 2011).

This study revealed that indigenous patients were three times more likely to get CRC at an earlier age compared to the Chinese patients, and this could be attributed by the interaction of multiple established risk factors associated with colorectal cancer, namely the modifiable and non-modifiable risks, thus leading to an earlier occurrence of CRC within this group (Wang et al., 2019; Jamal et al., 2020). An epidemiological study in the near future could help identify these risk factors and hence improve the community awareness towards this disease, leading to earlier screening for non-modifiable risks and avoidance of certain the modifiable risks (Wang et al., 2019). Having said that, an epidemiological study to identify risk factors could be costly and tedious, unless there is an international collaboration pertaining to assessing these risk factors in the indigenous population globally. Instead, it would be more helpful if the cost is used to start a national screening programme for CRC with an emphasis for earlier screening in the indigenous population (Wang et al., 2019). Furthermore, risk stratification tools also can be used to aid screening programme by identifying high risk individuals amongst the indigenous population (Wang et al., 2019).

In summary, the distinct clinico-pathological characteristics of Sabah’s indigenous population, in the context of local challenges - logistics, financial viability, and cultural norms – must be considered in strategic planning for optimal CRC care. This is important in order to make the pre-existing treatments accessible and future new treatments acceptable. 

In conclusion, indigenous populations in Sabah develop colorectal cancer at an earlier age, and present at more advanced stages compared to Sabahan Chinese. Nevertheless, CRC mortality in Sabah appears to be lower than national rates. Future work would focus on ascertaining the reasons for this, which are likely to be multifactorial. This has implications for screening and therapeutic strategic planning.

## Author Contribution Statement

Valan A and Roslani AC initiated, planned, and designed the study. Valan A had full access to all the data in the study and conducted the data acquisition, data analysis, interpretation of the findings and writing of the manuscript. Roslani AC supervised, reviewed and edited the manuscript. Najid F, Chandran P and Rahim AA were involved in the data collection in their respective hospitals. Chuah JA supervised the project. 
